# Measuring autistic traits in the general population: a systematic review of the Autism-Spectrum Quotient (AQ) in a nonclinical population sample of 6,900 typical adult males and females

**DOI:** 10.1186/2040-2392-6-2

**Published:** 2015-01-14

**Authors:** Emily Ruzich, Carrie Allison, Paula Smith, Peter Watson, Bonnie Auyeung, Howard Ring, Simon Baron-Cohen

**Affiliations:** Cambridge Intellectual and Developmental Disabilities Research Group, Department of Psychiatry, University of Cambridge, Douglas House, 18B Trumpington Road, CB2 8AH Cambridge, UK; Autism Research Centre, Department of Psychiatry, University of Cambridge, Douglas House, 18B Trumpington Road, Cambridge, CB2 8AH UK; MRC Cognition and Brain Sciences Unit, 15 Chaucer Road, Cambridge, CB2 7EF UK; Psychology Department, University of Edinburgh, 3 Charles Street, Edinburgh, EH8 9AD UK; NIHR CLAHRC for the East of England, Douglas House, 18B Trumpington Road, Cambridge, CB2 8AH England, UK; Cambridgeshire and Peterborough NHS Foundation Trust, Peterborough, CB21 5EF UK; CLASS Clinic, Cambridgeshire and Peterborough NHS Foundation Trust, Peterborough, CB21 5EF UK

**Keywords:** Autism, Autism-spectrum quotient, Autistic traits, Mean score, Sex differences, Systematic review

## Abstract

The Autism-Spectrum Quotient (AQ) is a self-report measure of autistic traits. It is frequently cited in diverse fields and has been administered to adults of at least average intelligence with autism and to nonclinical controls, as well as to clinical control groups such as those with schizophrenia, prosopagnosia, anorexia, and depression. However, there has been no empirical systematic review of the AQ since its inception in 2001. The present study reports a comprehensive systematic review of the literature to estimate a reliable mean AQ score in individuals without a diagnosis of an autism spectrum condition (ASC), in order to establish a reference norm for future studies.

A systematic search of computerized databases was performed to identify studies that administered the AQ to nonclinical participant samples representing the adult male and female general population. Inclusion was based on a set of formalized criteria that evaluated the quality of the study, the usage of the AQ, and the population being assessed.

After selection, 73 articles, detailing 6,934 nonclinical participants, as well as 1,963 matched clinical cases of ASC (from available cohorts within each individual study), were analyzed. Mean AQ score for the nonclinical population was 16.94 (95% CI 11.6, 20.0), while mean AQ score for the clinical population with ASC was found to be 35.19 (95% CI 27.6, 41.1). In addition, in the nonclinical population, a sex difference in autistic traits was found, although no sex difference in AQ score was seen in the clinical ASC population.

These findings have implications for the study of autistic traits in the general population. Here, we confirm previous norms with more rigorous data and for the first time establish average AQ scores based on a systematic review, for populations of adult males and females with and without ASC. Finally, we advise future researchers to avoid risk of bias by carefully considering the recruitment strategy for both clinical and nonclinical groups and to demonstrate transparency by reporting recruitment methods for all participants.

## Introduction

Autism was traditionally considered as a clinical condition distinct from the general population, but recent evidence suggests autistic traits are continuously distributed across the population [1–3]. From observed data of measured autistic traits, people with a diagnosis of an autism spectrum condition (ASC) - at least those who have average IQ or above - score at the extreme end of this distribution [4]. It may be that ‘syndromic’ forms of autism, which often entail comorbid learning disability (or below average IQ) and a known genetic mutation, are discontinuous with autistic traits in the general population, but here the focus is on the general population without learning disability. The Autism-Spectrum Quotient (AQ) is widely used in research and clinical practice to quantify autistic traits. The AQ was first developed as a self-report measure for adults [5] and subsequently as a parent-report measure for adolescents (aged 12 to 15 years) [6] and for children (aged 4 to 11 years) [7]. A toddler version also exists (Q-CHAT (Quantitative Checklist for Autism in Toddlers) [8]). The AQ has 50 items, which are divided into five subscales consisting of 10 items each that assess domains of cognitive strengths and difficulties related to ASC: communication, social skills, imagination, attention to detail and attention switching. While the AQ is not the only research tool used to measure autistic traits (for example, see the SRS (Social Responsiveness Scale [9]), it has several advantages over other measures, including subscales for both social and nonsocial aspects of behavior and cognition and a format that is brief, self-administered, and forced-choice.

The AQ was designed for adults with average IQ or above [5], who comprise at least 50% of the autism spectrum [10]. Individuals are instructed to respond to each of the 50 items with one of four responses: ‘definitely agree’, ‘slightly agree’, ‘slightly disagree’, and ‘definitely disagree’. Responses are scored using a binary system, where an endorsement of the autistic trait (either mildly or strongly) is scored as a +1, while the opposite response is scored as a 0, leading to a maximum score on the AQ of 50. An alternative scoring system has also been employed that uses a 4-point Likert scale [11]. AQ items are counterbalanced to avoid a response bias, so that half of the ‘agree’ responses and half of the ‘disagree’ responses endorse the autistic trait. The AQ includes questions about both ability and preference. The questionnaire is not suitable for individuals with low IQ, low verbal ability, or language impairment, as it relies on receptive understanding of the 50 questions.

The AQ was originally validated in 2001 in adult males and females with Asperger Syndrome (AS) and high-functioning autism (HFA), in scientists versus nonscientists in Cambridge University students, in winners of the mathematical Olympiad (because of the finding that autism may be genetically linked to an aptitude for ‘systemizing’ [12–14]), and nonstudent individuals drawn from the general population. This study found that the total AQ score and its five subscale scores are normally distributed and have demonstrated good test-retest reliability, good internal consistency [5], and that the measure has acceptably high sensitivity and specificity: at a cut-off score of 26, 83% of patients were correctly identified (sensitivity 0.95, specificity 0.52, positive predictive value 0.84, negative predictive value 0.78), while a cut-off score of 32 correctly identifies 76% of patients (sensitivity 0.77, specificity 0.74) [11, 15] when the AQ is used in a referred clinical sample.

These results indicate that the AQ is a sensitive measure of autistic traits in the general population, implying that traits reaching a clinical level in autism also exist to a lesser degree in nonclinical counterparts [5]. Within families, AQ score has shown heritability, which is in line with genetic evidence suggesting the heritability of autism [16]. Further, some (but not all) parents of children with autism show a subclinical set of characteristics or traits that index familiarity and/or genetic liability to autism [17, 18]. This is referred to as the ‘Broader Autism Phenotype’ (BAP). There is a consistent sex difference in mean AQ score, such that typical males score significantly higher than typical females, while people of both sexes with ASC score at the extreme high end of the scale, in line with the extreme male brain (EMB) theory of autism [19, 20].

The AQ is also widely referenced: a recent search of Google Scholar indicated that the original publication has been cited over 1,250 times. The present study reports the first large-scale systematic review of published AQ data over the last 13 years from adults with and without a diagnosis of ASC, in order to characterize the distribution of autistic traits in adult males and females and to contrast scores from clinical versus nonclinical samples. The specific goal is to establish a reliable mean AQ score in nonclinical controls, which can then be used as a guideline for researchers to define their control groups in future studies that compare people with and without a clinical diagnosis of ASC, as well as to other specially selected groups.

## Review

### Methods

#### Identification of relevant literature

Citation indexing databases Scopus, PubMed (Medline), PsycINFO, and Web of Science were queried for articles utilizing the AQ. Titles, abstracts and keywords were searched for (“autism quotient”) OR (“autis* spectrum quotient”) OR (“AQ” AND “autism”). Exploded MeSH terms were not used because of the narrow target of interest; studies were only considered if they explicitly mentioned the AQ. However, an additional search of Scopus and Web of Science was performed by which all peer reviewed journal articles citing the 2001 Baron-Cohen paper introducing the measure were retrieved. The two searches were merged; the citation search delivered 837 hits and the keyword search delivered 321 hits, 287 of which were retrieved by both methods.

Titles and abstracts and then full text articles, were reviewed. Inclusion criteria specified that the study had to include peer-reviewed empirical research (excluding all meta-analyses, literature reviews, book chapters, conference proceedings, *etcetera*.), be published in English, that the AQ had to be the 50-item AQ adult self-report (and not the AQ-Child, AQ-Adolescent or any of the abbreviated versions of the AQ), and that there was evidence that the English-language version of the AQ had been administered rather than any translations. The nonclinical participant sample had to include both males and females recruited from the population, with a mean age of 18 years or older.

**Figure 1:**
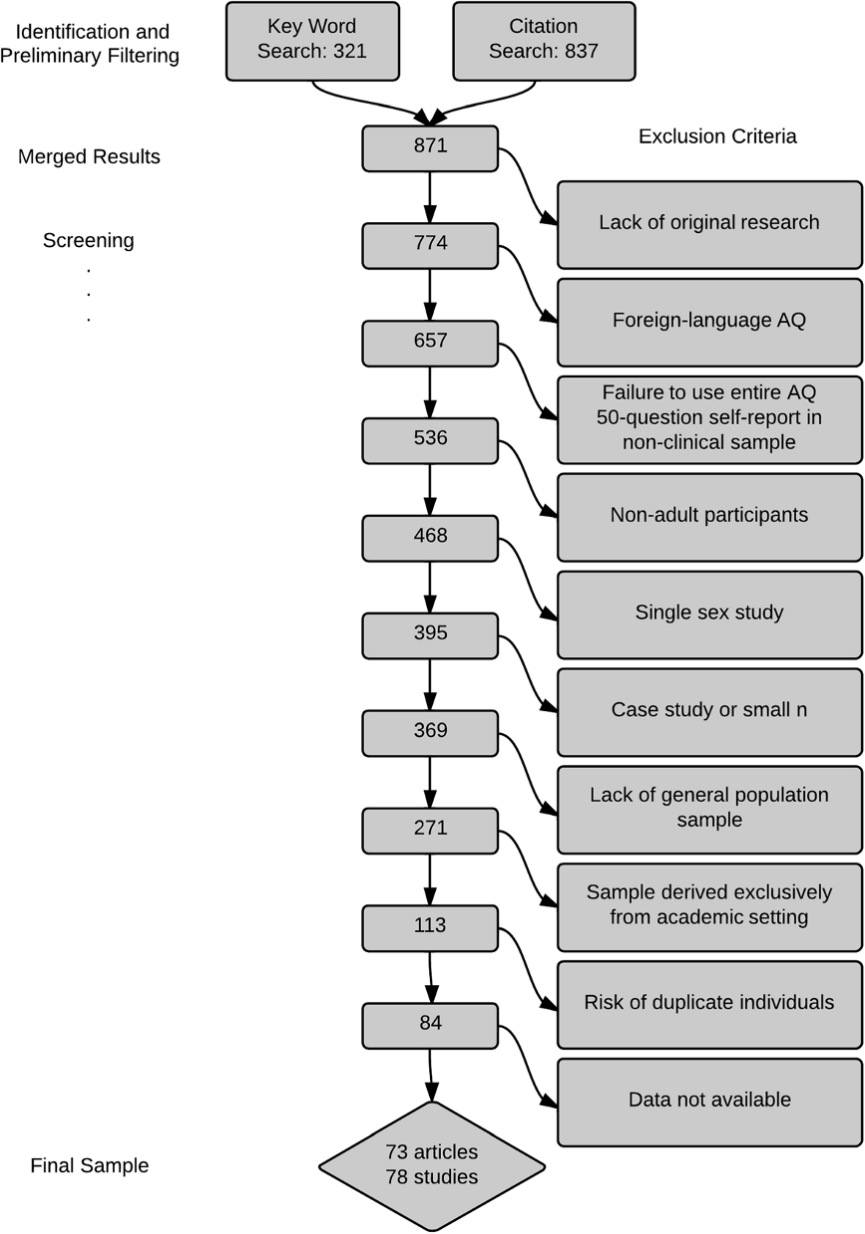
**Selection process for systematic literature review: post-database searches for English-language peer-reviewed research.**

Exclusion criteria were applied that assessed the quality of the study, the usage of the AQ, and the population being assessed. See Figure 1 for the selection process. Articles were excluded if they were case reports, studies containing fewer than 10 participants, or if the study specifically recruited participants who were immediate family members of an individual with ASC or patients with a particular mental or physical disorder or condition. In addition, due to findings from within the original AQ publication indicating the potential for academic disciplines to score more highly on the AQ, and in an effort to remove confounding variables such as age and education level, articles were excluded if participants had been recruited exclusively from within a university (though partial university recruitment was acceptable if authors indicated that an effort was made to recruit from outside the academic community). Articles were also excluded if an AQ cut-off score was imposed when delineating the control or nonclinical group. Where it was unclear whether an article met eligibility criteria, the article was retained. A number of research groups frequently recruit participants from the same database, which may potentially lead to the same individuals’ AQ scores being duplicated in analyses across more than one publication; to guard against the risk of duplication, articles from the same research group were assessed. If authors used similar phrasing in describing the recruitment process or explicitly stated that participants were drawn from the same database, the publication with the largest population group was included in analysis while the rest were excluded. Finally, several articles published in the same year by the same authors contained identical AQ scores and numbers of participants; in these rare cases, the earliest instance was included while the later publications were conservatively excluded.

In a number of instances, authors indicated that participants had completed the AQ but complete data sets were not reported. For 36 papers, authors were contacted for clarification or more information (11 articles were lost due to lack of a response). The deadline for data queries from authors and for literature searching was Monday, 14 July 2014. From the literature search and screening process 73 articles (reporting 78 independent studies) met the inclusion criteria.

#### Inter-rater agreement

The first author (ER) performed 100% of the literature search, quality assessment, and data extraction. In order to assess reliability of this process, approximately 10% of the results returned by the literature search were examined by authors CA, PS, and SBC. Each of the second reviewers received a random sample of 30 articles for evaluation (totaling 90). Where it was unclear whether an article met eligibility criteria, the article was discussed among the research team and if agreement was reached, it was retained for inclusion in the analysis. Initial percentage inter-rater agreement was respectively 97%, 90%, and 90%; after a resolution process, all disagreements between the lead author and the second raters were resolved in favor of the first author.

#### Extraction of data from included papers

The following information was recorded:

1. Number of participants, delineated by sex if reported
2. Mean and standard deviation of AQ score for males, females, and the sexes combined
3. Range of AQ score, if reported
4. Test for normality, if reported
5. Mean and standard deviation of participants’ age
6. Recruitment strategy, if reported
7. A comment on whether the study excluded individuals who were first-degree relatives of someone with a diagnosis of ASC
8. Margin of error and confidence intervals were calculated for each study by ER
9. Mean AQ score was recorded if the study included a matched sample of participants with ASC.

#### Data analysis

This systematic review aims to explore the distribution of a single variable - AQ score - in a large nonclinical population sample; therefore in this case, a meta-analysis (for effect sizes) is not possible. Data were imported into R [21] for systematic analysis. The mean of means was calculated by differentially weighting the reported values by sample size using weighted linear regression. In addition, the range of standard distributions, along with minimum and maximum values, was reported, and confidence intervals for reported average AQ scores were calculated. These values were also calculated for studies reporting separate male and female AQ scores, which were then compared using meta-analytic techniques. Finally, a small subset of studies (N = 9) reported that, in addition to taking a personal medical history, participants were only eligible to be considered a part of the nonclinical population group if they also had no first-degree relatives with ASC. For these studies, a separate mean of means for the AQ was also calculated. The focus of this study concerned average performance on the AQ, but standard deviation was also noted from eligible publications. From these scores, pooled variance was calculated.

While the primary focus of the review was to explore AQ scores in a nonclinical population sample, AQ score from the ASC sample for the selected papers was also noted where relevant. These scores were analyzed in the same method reported above. In addition to the quantitative approach described above, the papers that met criteria were subjected to a qualitative reading of the recruitment strategy for the nonclinical participant sample. This was in an effort to provide a description of the background for the participants included in analysis.

## Results

### Quantitative characterization of the Autism-Spectrum Quotient in a nonclinical population sample

**Figure 2:**
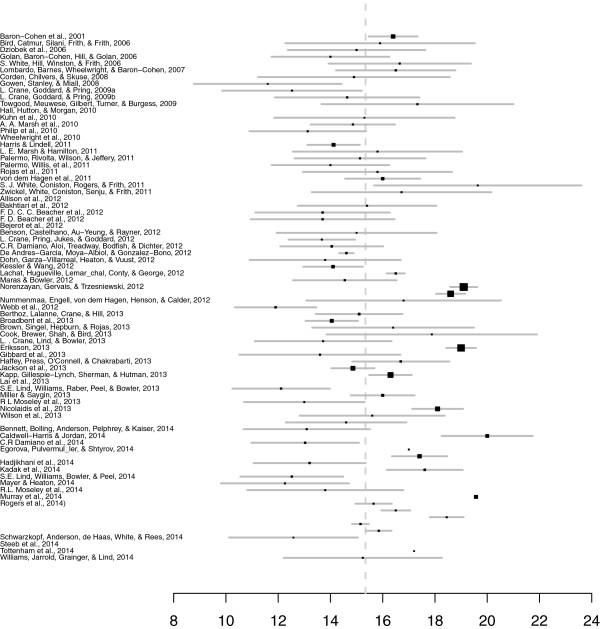
**Individual study overall Autism-Spectrum Quotient (AQ) means for nonclinical populations.** Bars indicate confidence intervals, point size scaled to the number of individuals in each study. Studies are ordered chronologically. From 2001 to 2011, the unweighted mean AQ score = 15.27 (SD = 1.73); from 2012 to 2014, m = 15.37 (SD = 2.12). Overall mean is indicated by the dotted line.

From a total of 73 articles reporting 78 studies that met eligibility criteria, data were recorded from 6,934 individual nonclinical participants. Table 1 describes the individual studies reviewed. See also inset plot for study AQ means and standard errors (Figure 2).

**Table 1:** **Articles selected for review**

	Nonclinical sample					Autism spectrum cases				
	Males	Females	Overall	Range	N (Females)	Males	Females	Overall	Range	N (Females)
	AQ m (SD)	AQ m (SD)	AQ m (SD) ^a^			AQ m (SD)	AQ m (SD)	AQ m (SD) ^a^		
[5]	17.8 (6.8)	15.4 (5.7)	16.4 (6.3)		174 (98)	35.1 (6.9)	38.1 (4.4)	35.8 (6.5)		58 (13)
[22]			15.9 (7.4)		16 (2)			37.5 (9.9)		16 (2)
[23]			15 (6)	6 to 30	20 (2)			38 (5)	28 to 46	21 (2)
[24]			14 (5.4)	6 to 26	22 (4)			38.5 (7.8)	16 to 49	22 (5)
[25]			16.65 (6.81)		24 (12)			34.63 (7.08)		16 (6)
[26]			16.5 (6.38)		30 (7)			33.93 (7.89)		30 (7)
[27]			14.9 (8.58)		21 (5)			37.1 (6.21)		21 (5)
[28]			11.6 (5)		12 (6)			36.7 (6)		12 (6)
[29]			12.53 (5.78)		18 (8)			35.28 (5.78)		18 (8)
[30]			14.64 (7.46)		28 (14)			34.93 (6.9)		28 (14)
[31]			17.33 (8.79)	2 to 42	22 (4)			35.86 (8.23)	17 to 48	21 (4)
[32]	18.81 (7.8)	17.21 (6.22)			35 (19)					
[33]			15.3 (6.1)		12 (*NA*)			30.6 (9.7)		12 (*NA*)
[34]			14.86 (4.03)		24 (12)					
[35]			13.13 (5.46)	6 to 29	23 (6)			34.39 (7.65)	21 to 46	23 (7)
[17]	17.7 (6.9)	13.1 (6.3)			988 (644)					
[36]			14.12 (5.78)		127 (84)					
[37]			15.8 (7.2)		19 (*NA*)			36.1 (8.7)		18 (*NA*)
[38]	19 (6.25)	12.15 (4.16)	15.13 (6.12)		23 (13)					
[39]			14 (4.74)		17 (10)					
[40]			15.8 (6.35)		19 (13)					
[41]			16 (7)	2 to 33	91 (53)					
[42]			19.64 (7.84)		15 (4)			37.06 (8.47)		16 (4)
[43]			16.72 (7.44)		18 (5)					
[44]	19 (7.87)	17.36 (7.99)		2 to 45	838 (509)	37.8 (7.8)	39.8 (6.0)		8 to 50	449 (209)
[45]			15.4 (5.9)		19 (3)			27.6 (5.7)		14 (2)
[46]			13.7 (7.43)		32 (16)			36.9 (7.05)		29 (14)
[47]			13.7 (7.7)		30 (15)			36.8 (7.1)		28 (13)
[48]	11.7 (5.4)	10.4 (4.2)			53 (25)	28 (9.4)	31.9 (7.9)			50 (24)
[49]			15 (5.63)		13 (7)			31.82 (9.59)		14 (4)
[50]			13.67 (2.76)		18 (6)			34.39 (5.26)		18 (6)
[51]			14.05 (6.19)	3 to 29	38 (4)					
[52]			14.61 (0.84)		35 (22)					
[53]			13.8 (5.9)		16 (3)					
[54]			14.1 (5.7)		96 (96)					
[55]			16.5 (1)		32 (16)					
[56]			14.55 (5.44)		29 (8)					
[57]			19.1 (7.03)		706 (445)					
			18.6 (6.04)		452 (229)					
[58]			16.8 (7.6)	7 to 33	16 (12)					
[59]			11.9 (4.5)	3 to 21	32 (3)			29.4 (7)	16 to 44	32 (2)
[60]			15.1 (5.8)	5 to 28	47 (18)			35.8 (5.9)	22 to 46	38 (38)
[61]			14.05 (5.8)		129 (83)			36.04 (7.13)		104 (46)
[62]			16.4 (6.11)		15 (9)			29.44 (8.6)		13 (4)
[63]			17.88 (8.21)		16 (4)			33.13 (10.09)		16 (1)
[64]			13.72 (5.67)		18 (5)			34.67 (6.89)		18 (5)
[65]			19 (7.1)		600 (383)					
[66]			13.6 (7.86)	4 to 37	25 (25)			36.6 (6.53)	22 to 49	25 (4)
[67]			16.69 (5.69)	7 to 31	36 (18)					
[68]			14.86 (6.22)	3 to 37	216 (*NA*)					
[69]			16.3 (7.7)		342 (79)			35.32 (7.69)		223 (155)
[70]	15.6 (6.9)	12 (4.8)			60 (30)	32.7 (7.3)	37.5 (6.7)			60 (30)
[71]			12.11 (5.03)		28 (7)			34.15 (8.61)		27 (6)
[72]			16 (5.1)	5 to 28	67 (52)					
[73]			13 (5)		18 (6)			34 (10)		18 (9)
[74]			18.1 (7.5)		228 (149)			36 (7.6)		209 (122)
[75]			15.6 (6)		18 (12)					
[76]			14.6 (5)		18 (2)			30.4 (4.6)		16 (3)
[77]			13.09 (5.79)		22 (13)					
[78]			20 (9.7)		119 (60)			36.4 (7.3)		70 (30)
[79]			13.03 (5.85)	2 to 26	31 (17)					
[80]			17 (7)	5 to 33	15 (10)					
[81]			17.41 (6.89)		163 (91)					
[82]			13.2 (6.1)		31 (3)			29.5 (7.5)		39 (3)
[83]	18.26 (4.49)	16.95 (4.67)	17.61 (4.57)		38 (19)					
[84]			12.52 (5.41)		29 (7)			34.44 (8.78)		27 (6)
[85]			12.26 (5.45)	3 to 21	19 (4)			35.16 (7.59)	21 to 45	19 (4)
[86]			13.8 (5.7)		17 (6)			37.3 (9.9)		14 (7)
[87]			19.57 (7.48)		134 (*NA*)					
[88]			15.65 (1.59)		20 (10)					
			16.5 (1.26)		20 (10)					
			18.45 (1.48)		20 (10)					
			15.15 (0.83)		26 (13)					
			15.85 (1.26)		26 (13)					
[89]	11.43 (4.2)	14.2 (4.5)	12.58 (4.36)	7 to 20	12 (5)	40.13 (6.77)	42.5 (4.42)	41.14 (5.8)		14 (6)
[90]	15 (6)	14 (5)			29 (16)	38 (13)	39 (13)			30 (16)
[91]			17.2 (5.2)		12 (4)					
[92]			15.24 (6.37)		17 (3)			35.59 (9.17)		17 (3)

Studies ordered chronologically; blank cells indicate data not reported (or for patients, AQ not administered to individuals with an autism spectrum condition). Overall AQ mean^a^ refers to combined male and female score.

Descriptive statistics (weighted mean AQ, range of standard deviation, total range, 95% confidence interval, number of studies, and number of participating individuals) are shown in Table 2. Overall SD reported from included studies ranged from 0.83-9.7. A pooled variance was calculated from the scores (σ^2^ = 31.26), leading to a pooled standard deviation of 5.59.

**Table 2:** **Descriptive statistics for selected articles**

	Nonclinical Sample ^a^			Nonclinical Sample - BAP excluded ^b^			Matched ASC Cases ^c^		
	Males ^d^	Females ^e^	Overall ^f^	Males ^d^	Females ^e^	Overall ^f^	Males ^d^	Females ^e^	Overall ^f^
Mean AQ	17.89	14.88	16.94	14.84	12.73	15.03	36.40	38.83	35.19
Range	11.4 to 19.0	10.4 to 17.4	11.6 to 20.0	11.7 to 18.3	10.4 to 17.0	11.9 to 17.6	28.0 to 40.1	31.9 to 42.5	27.6 to 41.1
SD Range	4.2 to 7.9	4.2 to 8.0	0.8 to 9.7	4.5 to 6.9	4.2 to 4.8	0.8 to 6.4	6.8 to 13.0	4.4 to 13.0	4.6 to 10.1
CI	16.7 to 19.1	13.3 to 16.5	16.4 to 17.4	7.0 to 22.7	4.9 to 20.6	13.0 to 17.1	33.1 to 39.7	36.3 to 41.4	34.5 to 35.9
N (studies)	10	10	72	3	3	7	6	6	39
N (participants)	872	1378	4931	77	74	174	363	298	1374

Nonclinical Sample^a^ describes reports from nonclinical samples. Nonclinical Sample – BAP excluded^b^ describes a subset of the previous sample, where it was specified that individuals from the BAP had been excluded. Matched ASC Cases^c^ describes reports from available matched autism spectrum cases. Statistics calculated for all available reported data (see also Table 1). Males^d^ and Females^e^ refer to available data reported by sex, while Overall^f^ refers to samples from studies reporting undifferentiated or combined male and female score. AQ: Autism-Spectrum Quotient; ASC: Autism spectrum conditions; BAP: broader autism phenotype; CI: confidence interval; N: number; SD: standard deviation.

**Figure 3:**
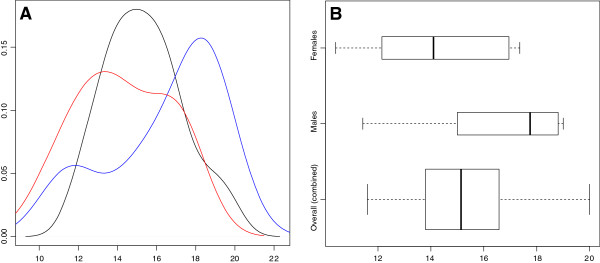
**AQ distributions for nonclinical populations. (A)** Kernel density estimates for unweighted Autism-Spectrum Quotient (AQ) distributions for nonclinical populations. AQ score on the x-axis and density on the y-axis. Male scores in blue, female scores in red, and combined scores in black. **(B)** Box plot of mean AQ scores for all studies. Box width scaled to reflect the number of studies included.

To compare the weighted mean AQ scores between males and females in studies that reported this information, a continuous random-effects model was used to find standard difference in means, SMD. There was a significant difference in scores between males and females: Hedges’ *g* = 0.40, *P* <0.001, z = 3.36. This holds true even if simple unweighted means are compared, though individual mean values are slightly reduced (Figure 3). A suggestion of bimodality was observed for males and females. However, previous observations of AQ scores indicate that there is a normal distribution within the population; likely this observation stems from the comparatively small number of data points used in this calculation (10 studies per group) or from the internal differences in study recruitment paradigms.

After initial selection criteria were applied, N = 9; [35, 40, 48, 52, 59, 62, 70, 83, 91]) studies were identified that excluded any individuals who might have the BAP from the typical group [17]. Table 2 presents the descriptive statistics for this set.

### Quantitative characterization of the Autism-Spectrum Quotient in a clinical sample

The 78 included studies were also examined for the presence of a matched clinical sample of individuals with a formal diagnosis of ASC. Of these, 43 studies contained data from 1,963 individuals with ASC (Table 1). Descriptive statistics for matched clinical cases are shown in Table 2. Overall SD reported from included studies ranged from 4.6 to 10.09. A pooled variance was calculated from the scores (σ^2^ = 39.27), leading to a pooled standard deviation of 6.27.

To compare the weighted mean AQ scores between clinical and nonclinical groups, a continuous random-effects model was used to find SMD. There was a significant difference in scores between these groups: Hedges’ *g* = 2.86, *P* <0.0001, z = 26.42, confirming that AQ scores are elevated in individuals with ASC. Contrasting with the findings reported for nonclinical controls, the SMD for males and females with ASC only reached a value of 0.33, which, while significant, indicates that males and females with ASC do not effectively differ in autistic traits as measured by the AQ; in fact, if anything, in this sample, the trend is reversed so that females self-report higher levels of traits.

### Trends in use of the Autism-Spectrum Quotient

In addition to reviewing the reported AQ scores, an effort was made to qualitatively assess AQ usage for included studies. Several trends were noted in administration and reporting of the full-scale AQ for adults. The majority of studies included in this review had recruited via newspaper adverts, employment agencies, email, post, and flyers. In many cases, the participants were also partially drawn from continuously maintained participant databases and research pools. There was also evidence of partial recruitment through hospitals and universities (though, as stated, studies were excluded where recruitment was exclusively within an academic community). In a number of instances, participants were recruited using publicly available online survey tools such as Amazon Mechanical Turk (M-Turk) and surveymonkey.com. Finally, several large studies were made possible through the use of birth cohorts, including the Raine Cohort (in Western Australia).

Few articles specified the precise inclusion and exclusion criteria for control participants, instead focusing primarily on the characterisation of the clinical group. While authors did routinely specify that control participants did not have a history of psychological or neurodevelopmental conditions, articles rarely reported having also excluded participants there was a family history of these. Studies also rarely reported testing the psychometric properties of the AQ or the normality of the distribution of AQ score. For instance, the mean was only reported alongside median in one instance [54], and there was also only one instance of a test for normality (Kolmogorov-Smirnov test [83]). There were occasional reports of other psychometric properties of the AQ, such as Cronbach’s alpha, establishing good internal consistency of the AQ.

## Conclusions

This is the first systematic review of the AQ, with several findings emerging. First, the mean AQ score in a typical sample drawn from a nonclinical population is approximately 17 (CI 16.4 to 17.4) (for those explicitly excluding BAP, the mean is approximately 15 (CI 13.0-17.1)), with a narrow confidence interval of one to two points. In addition, the mean AQ score in individuals with ASC is approximately 35, nearly 20 points above that found in the general population. Second, control males and females have significantly different average AQ scores, with males scoring higher, confirming earlier reports. Third, from 2001 onwards, there is considerable fluctuation in reported mean AQ scores, but scores have not appreciably drifted in one direction or another within the general population.

Several rationales were employed by researchers for using the AQ. Many of the studies administered the AQ not as a central variable correlated with the outcome measure, but as an accessory measure for characterizing the population or defining the experimental groups. Further, a number of articles used the AQ as a proxy for diagnosis, using the cut-off scores of either 32 or 26 to exclude individuals either from the clinical or from the nonclinical control group. (These articles were not included in the final analysis). However, caution is recommended when using the AQ in this way, as the AQ was designed to be a descriptive, rather than a diagnostic, measure of autistic traits. While, perhaps due to it being freely available, easy to administer, and widely precedented in the literature, the AQ is used as a screening instrument (such as for patients referred to a diagnostic clinic for a detailed assessment for ASC [93]), it has been argued that the AQ does not have the sensitivity and specificity for population screening with an eye to diagnosis [94–96]. This follows logically from the fact that the AQ is a brief self-report, reliant upon the individuals’ own self-awareness, and from the self-imposed limitations of age (16+) and IQ (85+). As discussed in the original publication, the AQ was developed from a theoretical understanding of autism, and therefore has not necessarily undergone the rigorous psychometric evaluation procedure that diagnostic screening tools must pass for inclusion in clinical practice. A more conservative use for the AQ is to segment the population into bands of autism phenotypes (broad, medium and narrow) as in the method of Wheelwright and colleagues [17], or as a descriptive quantitative measure of autistic traits.

Strengths of the current review include the exhaustive search criteria, especially the citation search for relevant papers, followed by the rigorous selection process. In addition, the total number of individuals (N = 8,897 clinical cases and nonclinical controls) examined by this review lends weight to the findings. The study was limited by a risk of bias, at the outcome level, the selection level and at the level of the review, though an effort was made to mitigate possible disproportionate effect of means from studies of varying samples through weighting by group size. Limitations also exist in the review procedure, in that each study included in the review was not judged for methodological rigor, rather a holistic evaluation was made of study methodology in an effort to report trends in items such as recruitment strategies, participant inclusion, and AQ data psychometric properties. Second, the number of participants from each study was relatively small (minimum N was set at 10); this is balanced by the large overall sample size derived from summing all studies together. More broadly, an ideal investigation of AQ score distribution would evaluate the raw data from each of the included studies in order to also measure data spread and subscale scores. However, this was not feasible for the current study. Finally, not every included article verified that the control group did not have ASC. Therefore there may have been incomplete information on how representative the demographic distribution of the nonclinical sample that make up this analysis may be.

We recommend that future researchers should think carefully when planning a recruitment strategy, both for nonclinical and clinical participants in order to be able to clearly define participants in each group. Furthermore, the field would greatly benefit if researchers better described the control participants, stating the method of recruitment in the methods. While healthy, typically-developing participants are often taken for granted, the considerable variability found in this review indicates that the method of recruitment of a ‘true’ representative sample - either of the general population or a specific patient group - may significantly impact results. This could have implications when examining group differences on dependent variables if the groups have not been carefully defined, potentially leading to attenuation of real groups differences.

We hope the current review holds value in the light of the considerable range of research types under which the AQ has been used. This dimensional approach to quantifying autistic traits has been found to correlate with a range of biological measures, including instances of brain activity [97], brain structure [98], social perception using gaze-tracking [42], prenatal testosterone [99], candidate genes and epigenetics [100]; clinical screening [44] and autism genetic risk [17]. Thus, although it is a self-report instrument, it correlates with a large number of more objective measures, suggesting that autistic traits are measurable aspect of personality, independent of the Big 5 [101].

Future research might consider a similar investigation of other versions of the AQ. Aside from the AQ-Adolescent and -Child, widely-used cross-cultural and foreign-language versions of the AQ exist, including translations into Chinese [102], Dutch [103], French [104], Italian [105], Japanese [106], Persian [107] and Polish [108], among others. On the whole, the results from studies that utilize these versions demonstrate analogous findings to those found using English-language versions of the AQ; however, validation by systematic review has not been done. In addition, a future study might attempt to undertake a whole population survey of autistic traits using the AQ, with more detailed information about the respondents collected in order to make stronger claims about generalizability. The underlying structure of taxa leading to AQ score distribution could be assessed using a number of modelling solutions, including latent class, taxometric, or factor mixture modelling. Perhaps, using these techniques in a population sample of individuals along the spectrum might help elucidate the apparent gap between clinical and nonclinical scores, despite the apparent continuity of autistic traits.

## Summary

The AQ continues to be a useful brief assessment instrument for measuring autistic traits in adults of normal intelligence. By determining the distribution of the AQ in the nonclinical population, the AQ can now be used more definitively to assess the extent to which other specialist populations exhibit autistic traits.

## Abbreviations

AQ: Autism-spectrum quotient
AS: Asperger syndrome
ASC: Autism spectrum condition
BAP: Broader autism phenotype
EMB: Extreme male brain
HFA: High-functioning autism
Q-CHAT: Quantitative checklist for autism in toddlers
SRS: Social responsiveness scale.
