# Erratum: Measuring autistic traits in the general population: a systematic review of the Autism-Spectrum Quotient (AQ) in a nonclinical population sample of 6,900 typical adult males and females

**DOI:** 10.1186/s13229-015-0038-8

**Published:** 2015-08-12

**Authors:** Emily Ruzich, Carrie Allison, Paula Smith, Peter Watson, Bonnie Auyeung, Howard Ring, Simon Baron-Cohen

**Affiliations:** Cambridge Intellectual and Developmental Disabilities Research Group, Department of Psychiatry, University of Cambridge, Douglas House, 18B Trumpington Road, CB2 8AH Cambridge, UK; Autism Research Centre, Department of Psychiatry, University of Cambridge, Douglas House, 18B Trumpington Road, Cambridge, CB2 8AH UK; MRC Cognition and Brain Sciences Unit, 15 Chaucer Road, Cambridge, CB2 7EF UK; Psychology Department, University of Edinburgh, 3 Charles Street, Edinburgh, EH8 9AD UK; NIHR CLAHRC for the East of England, Douglas House, 18B Trumpington Road, Cambridge, CB2 8AH England UK; Cambridgeshire and Peterborough NHS Foundation Trust, Peterborough, CB21 5EF UK; CLASS Clinic, Cambridgeshire and Peterborough NHS Foundation Trust, Peterborough, CB21 5EF UK

## Erratum

A correction should be made to the confidence intervals stated in the abstract, as they do not reflect those reported in the manuscript body. It was stated that "Mean AQ score for the nonclinical population was 16.94 (95 % CI 11.6, 20.0), while mean AQ score for the clinical population with ASC was found to be 35.19 (95 % CI 27.6, 41.1)." In fact, the first set of confidence intervals should read (95 % CI 16.4, 17.4) for the nonclinical sample, and the second should read (95 % CI 34.5, 35.9) for those with ASC.
